# Modulation of frontal EEG alpha oscillations during maintenance and emergence phases of general anaesthesia to improve early neurocognitive recovery in older patients: protocol for a randomised controlled trial

**DOI:** 10.1186/s13063-019-3178-x

**Published:** 2019-02-22

**Authors:** Amy Gaskell, Rebecca Pullon, Darren Hight, Jonathan Termaat, Gay Mans, Logan Voss, Matthias Kreuzer, Sebastian Schmid, Stephan Kratzer, Amy Rodriguez, Gerhard Schneider, Paul Garcia, Jamie Sleigh

**Affiliations:** 1 Department of Anaesthesiology, Waikato Clinical Campus, University of Auckland, Hamilton, New Zealand; 2 Department of Anaesthesia and Pain Medicine, Waikato District Health Board, Hamilton, New Zealand; 3 Department of Anaesthesiology and Pain Medicine, Inselspital, Bern University Hospital, University of Bern, Bern, Switzerland; 4 Center for Visual and Neurocognitive Rehabilitation, Atlanta VA Medical Center, Atlanta, GA USA; 5 Department of Neurology, Emory University School of Medicine, Atlanta, GA USA; 6 Department of Bioinformatics, Emory University School of Medicine, Atlanta, GA USA; 7 Department for Anesthesiology and Critical Care, Technische Universität München, Munich, Germany; 8 Department of Anesthesiology, Columbia University, New York, USA; 9 Neuroanaesthesia Division, Columbia University Medical Center, New York, USA; 10 New York Presbyterian Hospital, Irving, New York, USA

**Keywords:** General anaesthesia, Anaesthesia emergence, Delirium, EEG monitoring, Elderly

## Abstract

**Background:**

Postoperative delirium may manifest in the immediate post-anaesthesia care period. Such episodes appear to be predictive of further episodes of inpatient delirium and associated adverse outcomes. Frontal electroencephalogram (EEG) findings of suppression patterns and low proprietary index values have been associated with postoperative delirium and poor outcomes. However, the efficacy of titrating anaesthesia to proprietary index targets for preventing delirium remains contentious. We aim to assess the efficacy of two strategies which we hypothesise could prevent post-anaesthesia care unit (PACU) delirium by maximising the alpha oscillation observed in frontal EEG channels during the maintenance and emergence phases of anaesthesia.

**Methods:**

This is a 2 × 2 factorial, double-blind, stratified, randomised control trial of 600 patients. Eligible patients are those aged 60 years or over who are undergoing non-cardiac, non-intracranial, volatile-based anaesthesia of expected duration of more than 2 h. Patients will be stratified by pre-operative cognitive status, surgery type and site. For the maintenance phase of anaesthesia, patients will be randomised (1:1) to an alpha power-maximisation anaesthesia titration strategy versus standard care avoiding suppression patterns in the EEG. For the emergence phase of anaesthesia, patients will be randomised (1:1) to early cessation of volatile anaesthesia and emergence from an intravenous infusion of propofol versus standard emergence from volatile anaesthesia only. The primary study outcomes are the power of the frontal alpha oscillation during the maintenance and emergence phases of anaesthesia. Our main clinical outcome of interest is PACU delirium.

**Discussion:**

This is a largely exploratory study; the extent to which EEG signatures can be modified by titration of pharmacological agents is not known. The underlying concept is maximisation of anaesthetic efficacy by individualised drug titration to a clearly defined EEG feature. The interventions are already clinically used strategies in anaesthetic practice, but have not been formally evaluated. The addition of propofol during the emergence phase of volatile-based general anaesthesia is known to reduce emergence delirium in children; however, the efficacy of this strategy in older patients is not known.

**Trial registration:**

Australian and New Zealand Clinical Trial Registry, ID: 12617001354370. Registered on 27/09/2017.

## Background and rationale

There is current interest in the use of electroencephalographic (EEG)-guided administration of anaesthesia to prevent postoperative delirium and neurocognitive decline, prompted by findings of the association between low intraoperative EEG index values or burst-suppression patterns and postoperative delirium [1–3]. The underlying rationale is that reducing total anaesthetic dose and avoiding EEG suppression might improve neurocognitive outcomes. Prospective randomised clinical trials (RCTs) comparing titration of anaesthesia to processed EEG index ranges versus clinical signs have demonstrated reductions in postoperative delirium [1, 4]. However, the merit of targeting particular EEG index values or patterns, compared to purely avoiding the suppression pattern, is not known.

In our previous observational work of EEG patterns during maintenance and emergence from general anaesthesia, we have observed a variety of patterns in the EEG prior to waking [5, 6]. Although there is not a consensus among anaesthesiologists of what it means to be awake, we have started to distinguish between emergence and recovery from general anaesthesia. When we use the term ‘emergence from anaesthesia’ we are referring to the transition from surgical anaesthesia to a state with outward signs of cortical function (typically coordinated motor responses). One example would be eye contact in response to verbal or light tactile stimulation. Anaesthesia recovery is the transition from the end of emergence to normal pre-operative neurocognitive function. Defining when the actual complete period of anaesthesia has ended, and the subject has returned to a pre-anaesthesia baseline is difficult because a patient may be safe to discharge from the recovery room before all of the sedating effects of anaesthesia have subsided. Therefore, the complete recovery trajectory may go undocumented.

We have previously found that delirium (or incomplete neurocognitive recovery) in the recovery room seems to occur less often when patients display stronger alpha (7–17 Hz) oscillations in the frontal EEG during the emergence phase of anaesthesia [5, 6]. However, frontal EEG alpha power during maintenance of anaesthesia is negatively correlated with pre-operative cognitive impairment [7] and with advancing age [8–10], and this could explain the propensity for postoperative delirium. Nevertheless, the extent to which the EEG alpha oscillation is subject to intraoperative pharmacological manipulation has, thus far, gone largely unexplored.

Previous EEG-based protective strategies have focussed on targeting proprietary dimensionless index values, the meaning of which are not well understood. However, the EEG alpha oscillation is a specific hallmark of gamma-amino-butyric-acid (GABA)-ergic anaesthesia [11]. Marked loss of frontal EEG alpha activity can occur in response to noxious stimulation [12, 13] and the frequency and power of alpha oscillations are sensitive to changes in effect-site concentrations of volatile agents and opioids [8, 14]. We hypothesise that a maximal EEG alpha oscillation during general anaesthesia may represent an ideal anaesthetic state of both adequate anti-nociception and appropriate anaesthetic depth, which would represent a novel, individualised, anaesthetic strategy.

We also plan to investigate the efficacy of an alternative strategy; transition to propofol during the emergence phase of anaesthesia on the EEG patterns observed during emergence from anaesthesia, with the aim of preventing abrupt transitions in brain state. This intervention has been shown to reduce the incidence of emergence delirium in young children [15, 16] but has not been investigated adequately in older populations. As well as looking at EEG features and trajectories, we will evaluate the effect of these interventions on relevant clinical outcomes, particularly the incidence of delirium in the post-anaesthesia care unit (PACU).

### Aims

To ascertain whether the EEG patterns observed in an elderly population are amenable to modulation by titration of commonly used anaesthetic agents during the maintenance and emergence phases of anaesthesia. We will assess the efficacy of (1) titration of anaesthesia to maximise the observed frontal EEG alpha oscillation and (2) the addition of propofol as the main hypnotic during emergence in preventing PACU delirium.

### Hypotheses

1. The frontal observed EEG alpha oscillation is amenable to modulation by active titration of desflurane and opioid intraoperatively
2. Maximisation of the EEG alpha oscillation during the maintenance phase of anaesthesia promotes more alpha activity during subsequent emergence from anaesthesia
3. Supplementation with intravenously administered propofol promotes alpha activity during the emergence phase of anaesthesia
4. Alpha-dominant EEG emergence trajectories are associated with a lower incidence and/or severity of PACU delirium and possibly later adverse clinical outcomes

### Secondary objectives

1. To confirm or refute previously observed associations between maintenance and emergence EEG features and PACU delirium
2. To collect multichannel EEG recordings from a subset of patients to investigate associations between multichannel EEG markers and subsequent PACU delirium
3. To collect additional detailed data for possible confounding factors, such as physiological insults (hypotension, blood loss, hypothermia, urinary catheterisation), as risk factors for PACU delirium, and create a statistical predictive model
4. To investigate the prognostic utility of delirium scores and their subcomponents for subsequent adverse clinical outcomes and devise a specific PACU-delirium diagnostic tool
5. To detect abnormalities in speech-language function in PACU using an informal tool
6. To investigate whether the interventions have any effects on other early and late clinical outcomes such as early postoperative pain, recovery from surgery, serious adverse events and longer-term cognitive function

## Methods

### Sample selection and recruitment

Potential participants will be identified by screening of the operating theatre lists. In order to allow participants adequate time to decide if they wish to take part in the study we plan to approach patients in reasonable time before their surgery. This will be in person if they are admitted to the ward, or by a telephone call. The consent process will then be completed in person.

#### Inclusion criteria

Adults aged 60 years or over with capacity to provide informed consent who are undergoing elective non-cardiac surgery, which does not involve the head or neck, with planned volatile-based general anaesthesia of expected duration of at least 2 h

#### Exclusion criteria

Chronic pain with opioid requirement or concurrent use of enzyme inducers, e.g. carbamazepine, phenytoin, illicit substance use or excessive alcohol intake, refusal by patient or case anaesthetist responsible for patient’s care.

### Study design

**Fig. 1:**
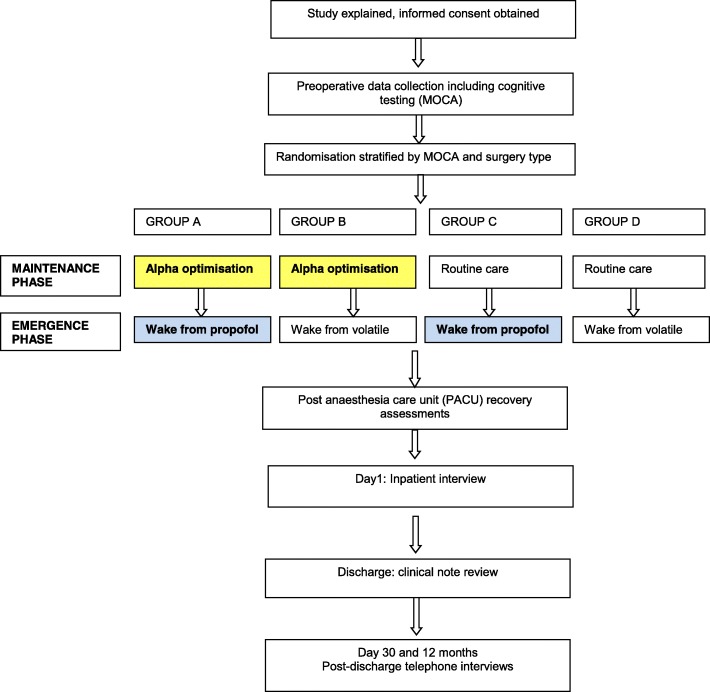
Study conduct flow sheet illustrating factorial design and data collection points

**Fig. 2:**
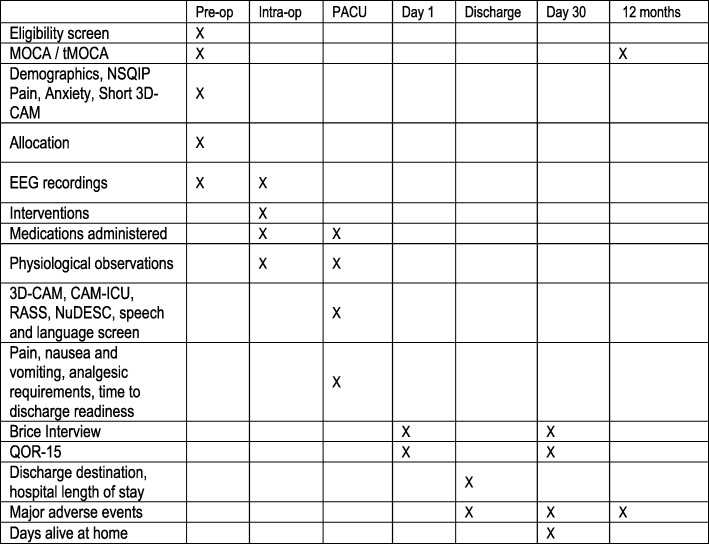
SPIRIT diagram demonstrating planned data collection

This is a prospective RCT using a 2 × 2 factorial design stratified by pre-operative cognitive score and surgery type. The 2 × 2 design means that each patient has a 50% chance of being allocated to each intervention. This will result in four randomisation groups, each with equal numbers of participants, namely (A) maintenance *and* emergence interventions, (B) maintenance intervention only, (C) emergence intervention only and (D) neither intervention (Fig. 1). Figure 2 illustrates the planned data collection at each time point in the study.

### Randomisation and blinding

Assignment of study participants to intervention groups will be performed using block randomisation [17], stratified by cognitive status (determined by pre-operative Montreal Cognitive Assessment (MOCA) score), surgery type (vascular, orthopaedic and general/other) and site. Pre-operative cognitive impairment is widely recognised as a risk factor for postoperative delirium and we have previously found that delirium risk appears to be lower for non-spinal orthopaedic procedures [6]. The researcher collecting intraoperative EEG data will perform the randomisation, shortly prior to commencement of the anaesthesia episode. The case anaesthetists will not be blinded to the interventions. Participants will be blinded to their interventions. Members of the research team responsible for recruitment, baseline data collection and clinical outcome data observers for the PACU scores and follow-up data collection will be blinded to randomisation groups and intraoperative events. For final analysis of the primary EEG outcomes, the raw EEGs will be de-identified so that the researcher conducting the analysis cannot identify the individual participant or the group allocation. We do not anticipate unblinding will be necessary.

### Anaesthesia conduct: all participants

To minimise potential confounding factors, the following recommendations will be made to the case anaesthetist for all groups:

1. Intravenous induction of anaesthesia with propofol and fentanyl (or equipotent dose of sufentanil). Maintenance anaesthesia with desflurane 0.5–1.2 minimum alveolar concentration (MAC) (age-adjusted). Desflurane has been chosen for consistency of offset, given its low lipid insolubility compared to sevoflurane and isoflurane
2. Fentanyl or sufentanil will be the only opioid analgesics permitted for both groups intraoperatively
3. Acceptable dose ranges of opioid and volatile anaesthesia to be decided and confirmed between the case anaesthetist and research team before starting the case
4. Avoidance of midazolam, total intravenous anaesthesia (TIVA), N_2_O, haloperidol, droperidol, tramadol, ketamine or clonidine intraoperatively as these may reasonably be expected to contribute to or prevent delirium or affect EEG parameters
5. Ondansetron, dexamethasone, metoclopramide and cyclizine are permitted intraoperatively, if deemed necessary for prophylaxis of postoperative nausea
6. Paracetamol/local anaesthesia/regional anaesthesia/non-steroidal anti-inflammatory drugs (NSAIDs) may be used freely by the case anaesthetist
7. Clinicians will be asked to avoid the burst suppression EEG pattern during maintenance phase of anaesthesia in all groups
8. The case anaesthetist has ultimate responsibility for the patient and will have discretion to overrule suggestions by the research team
9. Stimulation during the emergence phase of anaesthesia will be avoided as much as possible
10. Patient will be extubated on signs of return of consciousness, e.g. following loud repeated commands to open eyes/mouth

### First randomisation: maintenance phase of anaesthesia

Participants will be randomised to intraoperative oscillatory EEG alpha optimisation (Intervention 1) versus standard care (with burst suppression alert).

Intervention 1 involves real-time acquisition of oscillatory alpha power from the frontal EEG (recorded with the standard EEG electrode strip from the Entropy Module (GE Healthcare, Chicago, IL, USA) or the Bispectral Index (BIS) (Medtronic, Minneapolis, MN, USA) or other quantitative EEG monitor. An individual patient value for the maximal oscillatory alpha power ‘*Alpha Max*’ will be determined following induction of anaesthesia by variation of the end-tidal desflurane concentration prior to surgical incision. Effect-site concentrations of volatile and opioid will be estimated in real time to inform decisions around drug titration. During the intraoperative phase, we will aim to maximise oscillatory alpha EEG activity by individualised titration of desflurane and opioid. If alpha activity suddenly drops, the case anaesthetist will be advised to give a 0.5–1 mcg/kg bolus of fentanyl. Target end-tidal desflurane concentration will be adjusted according to real-time dose-response information. Typically, the concentration will be reduced in response to sustained loss of alpha activity; however, desflurane will not be reduced if quantitative EEG indices exceed a pre-defined threshold, to avoid awareness. For example, the State Entropy (SE) from the GE Entropy Module will be maintained below 60. The *Alpha Max* target will be adjusted intraoperatively if a new maximum is reached at any stage. The titrations will occur within prearranged limits agreed by the research team and the case anaesthetist and taking into account the clinical context, for example the degree of surgical stimulation.

For those patients randomised to standard care, anaesthesia will be conducted as per usual care by the anaesthetist, with quantitative processed EEG index values (e.g. State Entropy) and EEG waveforms visible as per the standardised protocol).

### Second randomisation: emergence phase of anaesthesia

Participants will be randomised to conversion to a propofol infusion for emergence from anaesthesia (Intervention 2) versus control (standard emergence from volatile anaesthesia).

This intervention will be achieved by infusion of propofol 400 mg/h and simultaneous reduction of inspired desflurane concentration during the final 10–20 min of surgery. Boluses of propofol may be given as required to keep acceptable quantitative EEG index values until surgery is complete. Total doses of propofol administered are likely to be in the range 1–3 mg/kg, which is comparable to the effective dose in children.

#### Primary outcomes

##### Intervention 1

- Oscillatory EEG alpha power during the maintenance phase of anaesthesia

##### Intervention 2

- Oscillatory EEG alpha power during the emergence phase of anaesthesia

Our primary trial outcomes are EEG outcomes (rather than clinical outcomes) because it is important firstly to determine whether the interventions, which are novel, do indeed have the anticipated effects on the intraoperative EEG parameters. Oscillatory alpha [12] is chosen as the outcome of neurophysiological interest because the total power in the frequency band is subject to general slowing of the EEG signal, and so the frequency of the alpha oscillation can drift outside the traditionally defined alpha frequency band.

#### Secondary outcomes

##### Intraoperative outcomes: EEG

- Maintenance phase
  - ○ Proportion of time with EEG alpha power > 70% of *Alpha Max* (the maximal EEG alpha power)
  - ○ Burst suppression duration
  - ○ Quantitative EEG indices
- Emergence phase
  - ○ EEG emergence trajectories
  - ○ Sequence and dynamics of slow wave (alpha and delta) activity and non-slow wave activity

We plan to evaluate the effectiveness of the intraoperative EEG alpha power-maximisation intervention by calculating the proportion of the maintenance phase during which the alpha oscillation is close to the maximum seen for that patient. This applies to the intervention group only since in the control group the maximal EEG alpha power will not be targeted. In addition to the primary outcome of oscillatory EEG alpha power, we will report on burst suppression and quantitative EEG indices. The EEG emergence trajectory patterns, in particular the presence or absence of abrupt state transitions, will also be categorised. A subset of patients will undergo multichannel EEG recording in addition to frontal EEG monitoring using locally available equipment, for later exploratory analyses.

##### Intraoperative outcomes: other

- Total opioid and desflurane administered intraoperatively
- Total muscle relaxant dose
- Hypotension (duration mean arterial pressure < 60 mmHg)

It is likely that the EEG alpha power-maximisation strategy will result in different patterns of administration of opioids and volatiles from the control group, which we will report. This could also result in differing muscle relaxant requirements and differences in intraoperative haemodynamic stability. In addition, we will collect feedback from the clinicians as to the acceptability of the protocol and the extent of any deviation from their usual care.

##### Immediate clinical outcomes: PACU

- Interval from extubation to fit-for-discharge status (achievement of Post Anaesthesia Recovery Score (PARS) score of 10/12 or more) from PACU
- PACU delirium
  - ○ 3D-CAM (and 3D-CAM-Severity)
  - ○ 3D-CAM features
  - ○ Nursing Delirium Screening Scale (NuDESC)
  - ○ Richmond Agitation-Sedation Score (RASS)
  - ○ Confusion Assessment Method for the Intensive Care Unit (CAM-ICU)
  - ○ Speech-language screen
  - ○ Requirement for pharmacological and non-pharmacological management of delirium
- PACU pain, nausea and vomiting
  - ○ Numerical rating score
  - ○ Opioid requirement
  - ○ Naloxone requirement

The ideal way to quantify neurocognitive recovery from anaesthesia is unclear so we intend to investigate a number of potential markers of both speed and quality of recovery. Extubation and fitness for discharge from PACU are easily recognised and readily recordable time points. A number of tools have been used to diagnose delirium in PACU, with variable performance [18, 19]. A *Diagnostic and Statistical Manual of Mental Disorders, fifth edition* (DSM-V) assessment by a trained physician or psychologist is usually considered the ‘gold standard’ for diagnosis of delirium; however, this is neither practical nor necessarily appropriate in the immediate post-surgical period; however, there is no single recommended PACU-delirium assessment tool [20]. The 3D-CAM [21] is a well-validated diagnostic instrument which we have found to be feasible in PACU in a small pilot study and this will be used as our main PACU-delirium outcome measure. In addition, we will perform the CAM-ICU, the NuDESC and a speech language screen to further cover the key neurocognitive domains. Our observations indicate that some patients experience language and/or cognitive communication deficits in PACU. Accordingly the inclusion of an informal speech-language screen may yield valuable information not typically obtained from delirium assessment tools. Tasks unique to this screen include category fluency, naming to definition and narrative production. Subsequent analysis of the later clinical outcomes will help to determine which features or scores are most important and of greatest prognostic value. Use of restraints or requirement of pharmacological treatment for PACU delirium will also be recorded. Since the EEG alpha power-maximisation strategy is hoped to maintain an optimal anti-nociceptive state intraoperatively, we will also examine whether there is any reduction in pain in PACU, using the numerical rating score and opioid requirements as the markers of this outcome. We will also record any requirement for naloxone reversal of opioid effect as a marker of excessive opioid administration.

##### Further clinical outcomes

- Quality of recovery (day 1 and day 30) – QOR-15
- Modified Brice Interview (day 1 and day 30)
- Hospital length of stay and discharge destination
- Days alive at home 30 days after surgery
- Serious adverse events (falls, unplanned high-dependency or intensive care unit admission, stroke or myocardial infarct, surgical site infection, hospital readmission and death) up to 12 months
- Cognitive status at 12 months (telephone Montreal Cognitive Assessment (tMOCA))

The QOR-15 is a validated score reflecting overall quality of recovery from anaesthesia and surgery [22, 23]. The Modified Brice Interview [24, 25] is used to check for awareness with recall. Days alive at home (DAAH) has been chosen as a validated patient-centred outcome measure that is readily quantifiable and reflects duration of hospitalisation, discharge destination and mortality [26]. Serious adverse events will also be recorded, from patient report and note review out to 12 months postoperatively to look at longer-term recovery and later serious complications. We will also conduct a tMOCA at 12 months as a final cognitive evaluation [27].

### Supplementary routine data collection

#### Baseline data

Use of hearing and visual aids will be recorded and these made available to the patient for the PACU assessments. We plan to collect standard demographic data including American Society of Anesthesiologists (ASA) score and planned surgery. Cognitive impairment is a known risk factor for the development of postoperative delirium [20] and will be used to stratify randomisation. The MOCA will be used to quantify cognitive status pre-operatively. In addition, some items from the 3D-CAM will be assessed at baseline to determine whether later errors in the 3D-CAM in PACU reflect a change from baseline performance. Pre-operative pain and anxiety will be recorded using the relevant questions from the QOR-15 questionnaire. The National Surgical Quality Improvement Programme (NSQIP) surgical risk calculator surgical complications risk [28] will also be calculated for each patient to potentially allow for risk adjustment in statistical analyses.

#### Intraoperative data

Intraoperative physiological data will be extracted from the operating room monitors using a custom-designed computer programme. Collected data will include heart rate, blood pressure, oxygen saturation, temperature, end-tidal carbon dioxide and volatile anaesthetic concentrations and the frontal EEG waveform and quantitative EEG indices. Additional EEG information will be obtained for a subgroup of consenting participants to investigate multichannel EEG features.

Effect-site concentrations of fentanyl and desflurane will be calculated and further EEG processing performed in real time. The oscillatory EEG alpha magnitude and dose-response relationships will be displayed to verify the oscillatory EEG alpha optimisation intervention.

The anaesthetic record will be photocopied so as to have a record of all administered medications. Pertinent event data, such as regional anaesthesia, estimated blood loss, total intravenous fluid volume administered and urinary catheterisation will be recorded routinely.

### Data management

Baseline data and clinical outcome data will be entered and managed using REDCap electronic data capture tools hosted at the University of Auckland, New Zealand. REDCap (Research Electronic Data Capture) is a secure, web-based application designed to support data capture for research studies, providing (1) an intuitive interface for validated data entry; (2) audit trails for tracking data manipulation and export procedures; (3) automated export procedures for seamless data downloads to common statistical packages and (4) procedures for importing data from external sources [29]. Intraoperative vital sign and EEG time-series data will be saved in real time to a computer used solely for data collection. At the end of a participant’s anaesthesia, the files will be transferred and stored on a secure server.

### Data safety monitoring

Risks to participants are expected to be minimal; the interventions being trialled are within the realms of current anaesthetic practice and the case anaesthetist will have the autonomy to digress from protocol in clinical interests of patients, e.g. if the doses suggested seem outside acceptable clinical practice. The primary risks that have been identified are light anaesthesia (with increased but still very low risk of awareness with recall) or deep anaesthesia and inadequate or excessive intraoperative analgesia. These risks will be measured and mitigated by the use of agreed dosage upper and lower limits, quantitative EEG monitoring, and interim analysis of PACU pain data. No formal interim analyses are planned; however, we will be taking informal feedback from clinicians throughout the trial and will review any unanticipated issues which arise.

A data safety monitoring committee will consist of two independent anaesthetists with research expertise. Any adverse events will be recorded and then categorised as to severity and the likelihood of the event having occurred as a result of the study. Adverse events will be reviewed regularly by the committee and the committee will make recommendations to the research team. Should any serious adverse events occur which are deemed due to the study itself, these will be communicated immediately to the committee to consider whether the trial should be suspended.

### Statistical analysis and power calculations

We intend to recruit 600 participants. The 2 × 2 factorial design will result in 300 patients assigned to the maintenance phase intervention and 300 to the emergence phase intervention (see Fig. 1). This will ensure that the study will be well-powered for the primary EEG outcomes and sufficiently powered to detect moderate-large effects in the secondary clinical outcomes. Our primary analyses will be intention to treat; however, per-protocol analyses will also be reported. For the primary outcome for the maintenance phase *Alpha Max* intervention, alpha power in groups A and B (Fig. 1) pooled together will be compared to groups C and D. For the primary outcome for the emergence phase intervention, groups A and C (Fig. 1) will be compared to groups B and D pooled. The crude difference will be reported as the primary outcome; however, an adjusted estimate will also be provided for the emergence outcome. This will include an interaction term for the maintenance phase intervention, since one might reasonably expect alpha power in the emergence phase to correlate with alpha power in the maintenance phase.

EEG analysis will be performed using customised script in MATLAB. Our previous summary data suggest that a mean oscillatory alpha power of 4.75 dB with standard deviation of 2.1 dB might be expected in a general surgical population. Therefore, an estimated 230 patients are required to have 95% power to detect an increase in oscillatory alpha of at least 1 dB (equivalent to ~ 20% relative increase in alpha power in response to the fentanyl) in the intervention group compared with the control group (two-tailed alpha 0.05, beta 0.05); thus, with a planned total of 300 patients per group we are comfortably overpowered for this outcome.

PACU delirium is our clinical outcome of primary interest, as a marker of neurocognitive recovery from anaesthesia. In our previous observational study, the incidence of CAM-ICU-diagnosed PACU delirium was 16% in those over 60 years and in a subsequent departmental audit, the incidence of PACU delirium diagnosed using the NuDESC tool was around 20% in those aged 65 years and over. Given that the study inclusion criteria requires > 2 h of anaesthesia, study participants will likely in general be having more extensive surgery and longer anaesthesia than those in the previous study and audit; therefore, we conservatively estimate a 20% incidence of PACU delirium in the control groups. From our observational work, the presence of EEG alpha has an odds ratio of approximately 0.5 for PACU delirium (unpublished data). Five hundred and thirty-two patients would be required to demonstrate a decrease in PACU delirium from 20 to 10% with 90% power (two-tailed alpha 0.05, beta 0.1, *χ*^2^ test). Thus, the study is powered to demonstrate strategies which have a large impact in preventing the development of delirium.

Almost certainly this study will also be sufficiently powered for minimum clinically important effects in some of the other secondary outcomes including delirium subcomponent features, days alive and out of hospital, and quality of recovery. We plan to recruit 600 patients to allow for loss to follow-up and a lower incidence of delirium than expected.

Continuous outcomes will be assessed using *t* tests and analysis of variance (ANOVA), ordinal with rank sum, and categorical outcomes will be assessed using the *χ*^2^ test, with significance set at *p* < 0.05. Non-linear mixed-effect modelling will be incorporated where appropriate to account for propensities such as: patient maximum alpha power, within-patient fentanyl and desflurane dose-response relationships and characteristics of the anaesthetic technique. Interactions between the two interventions are possible and will be examined. We also plan to carry out multivariate logistic regression to determine the major risk factors for the development of the features of PACU delirium, and receiver operator curve analysis to determine which elements of the early delirium screening tools are most closely associated with adverse clinical outcomes. *We anticipate performing further exploratory analyses in addition to those explicitly defined pre-operatively.*

### Ethical considerations

Older patients undergoing surgery constitute a potentially vulnerable population. Participants will be approached and consented by experienced research staff. We plan to only include participants who have given informed consent for surgery (or are expected to give their own consent for surgery) and, thus, would also be expected to be competent to consent to research. In addition, the research team will have discretionary ability to exclude participants should they feel that the patient is unable to sufficiently understand and consent to the study. However, it may be that some patients achieve scores much lower than is expected or normal for their age. It is important that this group is included in the study as they constitute a patient group that will potentially benefit most from the interventions.

Participants will be encouraged to discuss the study with any support people they wish and research staff will explain the study to support persons to aid decision-making. In order to assure that our study participants are capable of understanding the information provided in the informed consent, a further single question screen will be used to detect inadequate health literacy. ‘*Are you confident filling out medical forms by yourself*?’ has been used previously to detect poor health literacy [30]. If the answer to this question is ‘no’ or the pre-operative MOCA score is very low (less than 15/30) and it appears that the patient is unable to fully understand the implications of taking part in the study, they will be excluded from the study and the clinical team will be informed. We will check during the consent process the participant’s wishes about information disclosure should we detect early cognitive impairment and these wishes will be followed. The usual clinical standard of preservation of confidentiality will be preserved; data will not be published or shared in any such way that would reasonably be expected to identify individuals.

## Discussion

As far as we are aware, this will be the first RCT whereby anaesthesia is titrated to the observed frontal EEG alpha oscillation, rather than quantitative EEG index values. Furthermore, we hope to establish the degree of association between intraoperative alpha activity and PACU delirium, which is unclear at present.

Features of anaesthesia and delirium overlap considerably and it is difficult to define when anaesthesia emergence ends. Current nomenclature can be quite confusing with terms such as emergence agitation, hypoactive, hyperactive, PACU delirium and postoperative delirium employed for somewhat similar states, which also fluctuate with time. The syndrome we are evaluating may in fact better be considered a delayed or incomplete neurocognitive recovery from anaesthesia. This clinical relevance of this syndrome is unclear and it is relatively sparsely represented in the literature; however, the small body of existing evidence suggests that PACU delirium is associated with multiple adverse clinical outcomes [31]. With this study we hope to establish whether this is simply a result of existing patient and surgical factors or if optimisation of anaesthesia might be protective.

The optimal assessment tool to measure delirium in the PACU environment is unclear. We have elected to use the 3D-CAM score as our main clinical outcome measure but we will also take the opportunity to evaluate and compare some other candidate scores. A secondary objective of the study will be to evaluate which features of delirium are most closely associated with later adverse outcomes. A recent consensus guideline [20] has recommended routine screening of patients for delirium prior to discharge from the recovery room; however, it does not specify a particular tool. Ideally the assessment tool should identify patients at risk of subsequent deterioration and adverse clinical outcomes. We plan to evaluate the performance of 3D-CAM in this regard, and to possibly create a tool specifically for an immediate post-anaesthesia care episode.

This study has been approved by the New Zealand Health and Disability Ethics Committee ref. 17/NTA/56 and has local intstitution approval at Waikato Hospital. Ethics/IRB approval and local institutional approval will be obtained prior to commencement of the study at other sites. Recruitment began in February 2018 at our primary site with plans for at least two international collaborating sites to join. As of October 2018, we have recruited 60 patients. Data collection is expected take 3–4 years. Dissemination plans include presentations at scientific conferences and publication in peer-reviewed journals.

### Study sites

The study will be carried out across three sites with possible later inclusion of additional sites:

- Waikato DHB Department of Anaesthesia, Waikato, New Zealand
- Technical University of Munich, Munich, Germany
- Columbia University Medical Center, Ithaca, NY, USA
